# Preparation and Characterization of Nano-CaCO_3_/Ceresine Wax Composite Shell Microcapsules Containing E-44 Epoxy Resin for Self-Healing of Cement-Based Materials

**DOI:** 10.3390/nano12020197

**Published:** 2022-01-07

**Authors:** Wei Du, Erwang Li, Runsheng Lin

**Affiliations:** 1 School of Material Science and Chemical Engineering, Ningbo University, Ningbo 315211, China; erwang_li@163.com; 2 State Key Laboratory of Silicate Materials for Architectures, Wuhan University of Technology, Wuhan 430070, China; 3 Department of Integrated Energy and Infra System, Kangwon National University, Chuncheon-si 24341, Korea; linrunsheng@kangwon.ac.kr

**Keywords:** self-healing, microcapsules, cement-based materials, nano-CaCO_3_, nanoindentation

## Abstract

As an intelligent material, microcapsules can efficiently self-heal internal microcracks and microdefects formed in cement-based materials during service and improve their durability. In this paper, microcapsules of nano-CaCO_3_/ceresine wax composite shell encapsulated with E-44 epoxy resin were prepared via the melt condensation method. The core content, compactness, particle size distribution, morphologies, chemical structure and micromechanical properties of microcapsules were characterized. The results showed that the encapsulation ability, mechanical properties and compactness of microcapsules were further improved by adding nano-CaCO_3_ to ceresine wax. The core content, elastic modulus, hardness and weight loss rate (60 days) of nano-CaCO_3_/ceresine wax composite shell microcapsules (WM2) were 80.6%, 2.02 GPA, 72.54 MPa and 1.6%, respectively. SEM showed that WM2 was regularly spherical with a rough surface and sufficient space inside the microcapsules to store the healing agent. The incorporation of WM2 to mortar can greatly improve the self-healing ability of mortar after pre-damage. After 14 days of self-healing, the compressive strength recovery rate, proportion of harmful pores and chloride ion diffusion coefficient recovery rate increased to 90.1%, 45.54% and 79.8%, respectively. In addition, WM2 also has good self-healing ability for mortar surface cracks, and cracks with initial width of less than 0.35 mm on the mortar surface can completely self-heal within 3 days.

## 1. Introduction

The durability of concrete has received much attention in the field of construction materials due to the need for extended service life [1,2]. However, cracks are considered to be one of the most important factors affecting the durability of cement-based materials during service, and their appearance is inevitable [3,4]. The extension of cracks can lead to deterioration of cement-based materials, thus reducing their service life [5]. However, the “after-the-fact” nature of conventional maintenance suggests that cracks can only be repaired after they have occurred, which not only increases costs but also requires advanced techniques to accurately detect and locate internal cracks [6]. To meet this requirement, self-healing cement-based materials aimed at automatic crack repair and service life extension have attracted the interest of many researchers [7,8].

The current self-healing technologies of cement-based materials mainly include: mineral self-healing technology [9], microbial self-healing technology [10] and microencapsulated self-healing technology [11]. The main mechanism of mineral self-healing technology is to mix mineral admixture into cement-based materials, and when there is water infiltration into cracks, the mineral admixture with special composition reacts with the calcium hydroxide generated by hydration inside the cement-based materials in a pozzolanic reaction or swelling to produce CaSiO_3_ gel and other crystals, thus achieving the purpose of healing the crack [12]. However, mineral admixtures have limited effectiveness in healing cracks in cement-based materials with widths exceeding 0.15 mm [13]. Microbial self-healing technology refers to the addition of microorganisms to cement-based materials to promote the production of some water-insoluble organic or inorganic compounds (e.g., CaCO_3_) through the metabolism or respiration of microorganisms for the purpose of healing cracks and filling pores [14]. However, microbial self-healing technology still has some problems to be solved, e.g., the pores of cement-based materials will decrease with age, the activity of extruded bacterial spores will decrease, and the self-healing ability will be reduced. The improper selection of microbial nutrient solution can reduce the mechanical properties and durability of cement-based materials [15]. Microencapsulated self-healing technology involves the direct incorporation of microcapsules (<1000 μm in diameter) containing healing agents (core materials) into the cement-based materials [16]. When triggered by cracks or changes in the surrounding environment, the outer shell of the microcapsule ruptures, and the healing agent is released to heal cracks or defects in the cement-based materials [17]. Compared with the above self-healing technologies, microencapsulated self-healing technology has good application prospects due to its advantages, such as environmental adaptability and fast healing rate [18].

From the proposal of microencapsulated self-healing technology to the present, many researchers have conducted numerous studies. Cailleux [19] prepared microcapsules with tung oil, Ca(OH)_2_ and epoxy resin as core materials and gelatin as the shell and studied the presence of microcapsules in a strong alkaline environment to evaluate the self-healing ability of microcapsules. The results showed that the microcapsules containing tung oil had a good healing effect on the mortar specimens. Li [20] investigated the effect of microcapsules with diglycidyl ether of bisphenol A epoxy resin as healing agent and polystyrene–divinylbenzene (St-DVB) as shell material on the self-healing performance of cement-based materials. Li indicated that the best recovery of mechanical properties of specimens (60% pre-load damage) under standard curing was achieved when microcapsules were mixed at 2% of the cement mass. Dong [21] studied the effect of urea-formaldehyde (UF) resin containing E-51 epoxy resin microcapsules on the self-healing properties of cement-based materials, and he found that the mortar containing microcapsules healed cracks as efficiently as 45.6%, and the recovery rates of compressive strength and permeability were 13.0% and 19.8%, respectively. Although the microencapsulated self-healing technology has good application prospects and good research progress, it still faces the following problems to be solved: (1) Currently, microcapsules are prepared by chemical methods, such as in situ polymerization or interfacial polymerization, which carry the risk of producing chemical contamination; (2) The microcapsules are difficult to rupture under crack tip stress due to the high mechanical strength of the thermosetting polymeric shell (St-DVB, UF, etc.) [22].

To address the above problems, microcapsules (microcrystalline wax containing E-51 epoxy resin) have been prepared and applied to cement-based materials for self-healing in previous research work [23]. However, although these microcapsules rupture more easily under external forces, the mechanical properties of the shell material are relatively low and the risk of rupture during mixing process is high. Moreover, in addition to mechanical properties, leakage of the core material (healing agent) is also a major factor affecting the self-healing ability of microcapsules. Recently, several researchers have synthesized shell materials by adding nanomaterials to improve the mechanical properties and compactness of microcapsules. Jiang [24] studied the synthesis of nano-Al_2_O_3_ modified poly(methyl methacrylate-co-methyl acrylate) coated paraffin. Li [25] prepared microcapsules by using nano-Fe_3_O_4_ mixed with paraffin to coat isocyanate. These studies showed that it is feasible to improve the mechanical properties and compactness of microcapsules by incorporating nanomaterials into the shell material.

In this paper, microcapsules of nano-CaCO_3_/ceresine wax composite shell and E-44 epoxy resin healing agent were prepared via the melt condensation method in order to improve the micromechanical properties and compactness of microcapsules. The core content and compactness of microcapsules were measured. The particle size distribution, morphology, chemical structure and micromechanical properties of the microcapsules were characterized. The microcapsules were mixed into the mortar, and the pore size distribution, compressive strength recovery rate and chloride ion diffusion coefficient recovery rate of the pre-damaged mortar containing microcapsules after self-healing were measured. The self-healing ability of microcapsules on mortar surface cracks was evaluated.

## 2. Materials and Methods

### 2.1. Materials

Ceresine wax (melting point: 93–95 °C) was obtained from Sinopharm Chemical Reagent Co., Ltd. (Shanghai, China). E-44 epoxy resin and *N*,*N*-dimethylformamide (DMF) were purchased from Ningbo Yongchuan Biotechnology Co., Ltd. (Ningbo, China). Nano-CaCO_3_ (particle size: 20 nm) was provided by Tianjin Yuoer Co., Ltd. (Tianjin, China). The 2-ethyl-4-methylimidazole, perfluorotributylamine and acetone substances were supplied by Beijing Letai Chemical Reagent Co., Ltd. (Beijing, China). Portland cement (P.O 42.5) was bought from Anhui Conch Cement Co., Ltd. (Wuhu, China). The chemical composition of cement is shown in Table 1. The CEN Standard sand (ISO standard sand) was used, which is siliceous, particularly its finest fractions. The particle size distribution of the sand is shown in Table 2.

### 2.2. Preparation of Microcapsules

**Figure 1:**
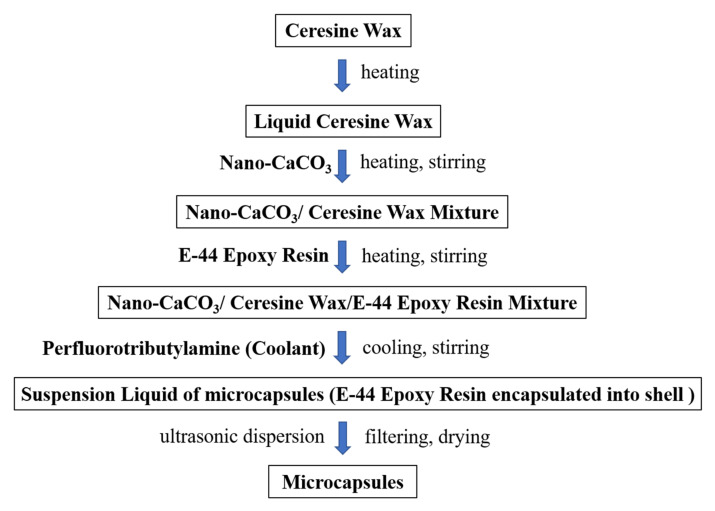
Schematic diagram of the microcapsule preparation process.

The microcapsules were prepared via the melt condensation method, which is a physical method with a simple process, high preparation efficiency and no chemical reactions during the preparation. First, the raw materials for the shell (1. ceresine wax; 2. nano-CaCO_3_/ceresine wax) were melted and dispersed; then, the E-44 epoxy resin, which was diluted with N, N-dimethylformamide (DMF), was added into the mixture of the shell with continuous heating and stirring, where the amount of DMF was 15% of the weight of E-44 epoxy resin; perfluorotributylamine (coolant) was added to the mixture, so that the temperature of the mixture was rapidly reduced to below the melting point of ceresine wax to obtain a microcapsule suspension; the microcapsule suspension was shaken with ultrasonic waves for 30 min; finally the microcapsules were filtered and dried (70 °C, 24 h). Table 3 illustrates the preparation parameters and shell/core weight ratios of microcapsules. Figure 1 summarizes the preparation process of WM2.

### 2.3. Preparation of Mortars

To test the mechanical properties and impermeability, two sizes of mortars were prepared. First, cement, sand and microcapsules were mixed in a mortar mixer for 1 min; then water and 2-ethyl-4-methylimidazole were added to the mortar mixer, and mixing was continued for 1 min. The 2-ethyl-4-methylimidazole substance was added at 20% of the weight of the diluted epoxy resin. Prismatic samples with casting dimensions of 70.7 mm × 70.7 mm × 70.7 mm were used for mechanical property testing. Cylindrical samples with dimensions ϕ100 mm × 50 mm were used for the chloride ion diffusion test. Finally, the mortar was demolded after 24 h and moved to the standard curing room (20 ± 2 °C, relative humidity ≥ 95%) for 28 days of curing. The mortar formulations are shown in Table 4. The microcapsules used for mortar preparation were left in air for 60 days.

### 2.4. Measurement and Characterization of Microcapsules

#### 2.4.1. Core Content

A certain number of microcapsules were randomly selected to determine the core content of microcapsules. These microcapsules were first weighed. The microcapsules were cut with a blade, and the encapsulated epoxy resin was rinsed off with acetone. The residual shell material was dried in an oven set at a temperature of 60 °C for 12 h and then weighed. The core content of microcapsules was calculated by Equation (1):

(1) $$\theta \;=\;\frac{M\;-\;m}{M}\;\times \;100\%$$

where θ is the core content of microcapsules (%), M is the weight of microcapsules (g) and m is the weight of the residual shell material (g).

#### 2.4.2. Compactness of Microcapsules

The prepared microcapsules were selected and weighed directly. Then, the weighed microcapsules were stored in a curing cabinet (25 °C, 50% RH). The weight loss of microcapsules was monitored after 1, 2, 4, 7, 14, 30, 45 and 60 days, respectively. The weight loss rate of microcapsules was calculated by Equation (2):

(2) $$\varphi \;=\;\frac{M_{1}\;-\;M_{2}}{M_{1}}\;\times \;100\%$$

where φ is the weight loss rate of microcapsules (%), M_1_ is the original weight of microcapsules (g) and M_2_ is the present weight of microcapsules (g).

#### 2.4.3. Size Distribution

A Mastersizer 2000 laser particle size analyzer was used to determine the average particle size and particle size distribution of the microcapsules (Malvern Instruments Ltd., Malvern, UK). Prior to testing, the microcapsules were placed in an oven at 70 °C for 12 h and then dispersed in anhydrous ethanol in the analysis chamber.

#### 2.4.4. Morphology

The S-4800 scanning electron microscopy (SEM) was applied to observe the morphology of microcapsules (Hitachi, Tokyo, Japan). Prior to SEM observation, some microcapsules were cut with a sharp blade, cleaned with acetone and dried. The gold film was sprayed on the surface of the intact and broken microcapsules, and a voltage of 3 kV was applied.

#### 2.4.5. FTIR Spectrum

The ceresine wax, nano-CaCO_3_, E-44 epoxy resin, DMF and microcapsules were characterized by FTIR spectroscopy (Nexus, Thermo Nicolet Corporation, Madison, WI, USA). The ceresine wax, nano-CaCO_3_ and microcapsules were mixed with potassium bromide and compressed into sheets. E-44 epoxy resin and DMF were brushed on potassium bromide flakes. The test conditions were set as follows: 64 scans, 4 cm^−1^ resolution, 4000~400 cm^−1^ test range.

#### 2.4.6. Nanoindentation Test

The nanoindentation tests were performed by a TriboIndenter (TI-900, Hysitron, Minneapolis, MN, USA) and fitted with a Berkovich tip. The specific testing process is described in detail in the Supplementary Materials.

### 2.5. Self-Healing of Pre-Damaged Mortars

#### 2.5.1. Compressive Strength

After 28 days of standard curing, six specimens from each group were taken for mortar compressive strength testing, and the average value was taken to determine the compressive strength of the mortar, expressed in f_a_. The mortar was loaded to 60%f_a_ to obtain pre-damaged specimens. The pre-damaged specimens were then placed in the laboratory (25 °C, 50% RH) for 1, 4, 7 and 14 days of self-healing. At last, the specimens were reloaded, and the compressive strength recovery rate was calculated by Equation (3):

(3) $$\delta \;=\;\frac{f_{n}}{f_{a}}\;\times \;100\%$$

where δ is the compressive strength recovery rate of the mortar, f_a_ is the initial compressive strength of the mortar and f_n_ is the compressive strength of the mortar after n days of self-healing.

#### 2.5.2. Pore Size Distribution

The pore size distribution of the mortar was investigated using a nuclear magnetic resonance spectrometer (MesoMR25, Suzhou Newman Analytical Instrument Co., Ltd., Suzhou, China). The specific testing process is described in detail in the Supplementary Materials.

#### 2.5.3. Impermeability

After 28 days of standard curing, the chloride ion diffusion coefficients of the specimens were determined according to the standard NT Build 492 [26]. The chloride ion diffusion coefficient of the pre-damaged control mortar (60%f_a_) was expressed as CCM60. The pre-damaged specimens were self-healed for 1, 4, 7 and 14 days, respectively, and the recovery rate was calculated by Equation (4):

(4) $$\varepsilon \;=\;-\frac{\varepsilon_{2}\;-\;\varepsilon_{1}}{\varepsilon_{1}}\;\times \;100\%$$

where ε is the chloride ion diffusion coefficient recovery rate (%), ε_1_ is the chloride ion diffusion coefficient of the control mortar after 60%f_a_ pre-load (10^−12^ m^2^/s) and ε_2_ is the chloride ion diffusion coefficient of the pre-damaged mortar after self-healing (10^−12^ m^2^/s).

### 2.6. Self-Healing of Surface Cracks

**Figure 2:**
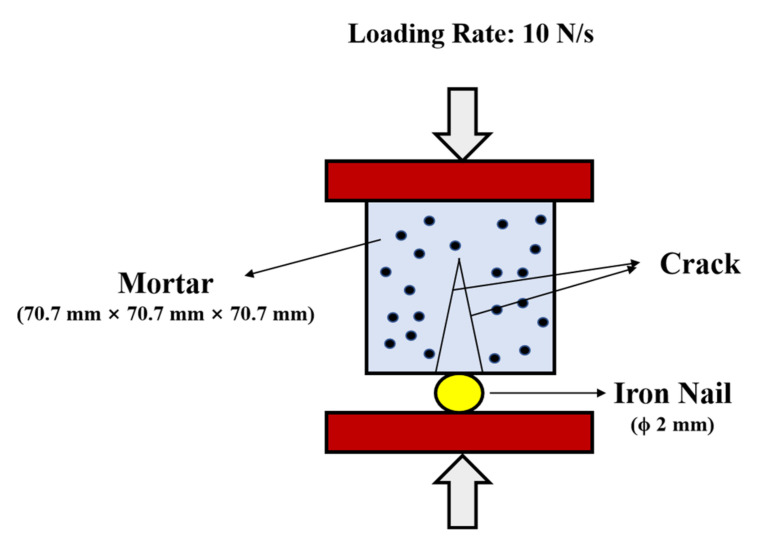
Schematic diagram of the test for mortar pre-cracking.

After 28 days of standard curing, the mortar specimens were pre-cracked at a constant rate of 10 N/s using an automatic compressive apparatus. A schematic diagram of this pre-cracking process is shown in Figure 2. The initial crack width was measured by a crack testing apparatus (PTS-E40, Wuhan Botest Instruments Co., Ltd., Wuhan, China). Three days later, the width of the crack was measured again.

## 3. Results and Discussion

### 3.1. Core Content of Microcapsules

Table 5 shows the core content of ceresine wax containing E-44 epoxy resin microcapsule (WM1) and nano-CaCO_3_/ceresine wax containing E-44 epoxy resin microcapsule (WM2). The core contents of WM1 and WM2 were 75.4% and 80.6%, respectively. This is mainly due to the different viscosities of the shell materials of these two microcapsules. As can be seen from Table 3, the viscosity of ceresine wax was relatively low (5.5 mPa·s) at the preparation temperature (130 °C), and the material can be uniformly dispersed after melting by heat. After adding the coolant, the particle size of the formed microcapsules was slightly smaller, resulting in a small amount of healing agent (E-44 epoxy resin) remaining outside the shell, therefore the core content of the microcapsule was relatively low. The viscosity of the nano-CaCO_3_/ceresine wax mixture (130 °C, 35 mPa·s) was higher than that of ceresine wax after the addition of nano-CaCO_3_ to ceresine wax, which reduced the dispersibility of the material. The addition of nano-CaCO_3_ also reduced the solidification time of the shell and improved the encapsulation ability, which resulted in a higher core content of the microcapsules.

### 3.2. Compactness of Microcapsules

**Figure 3:**
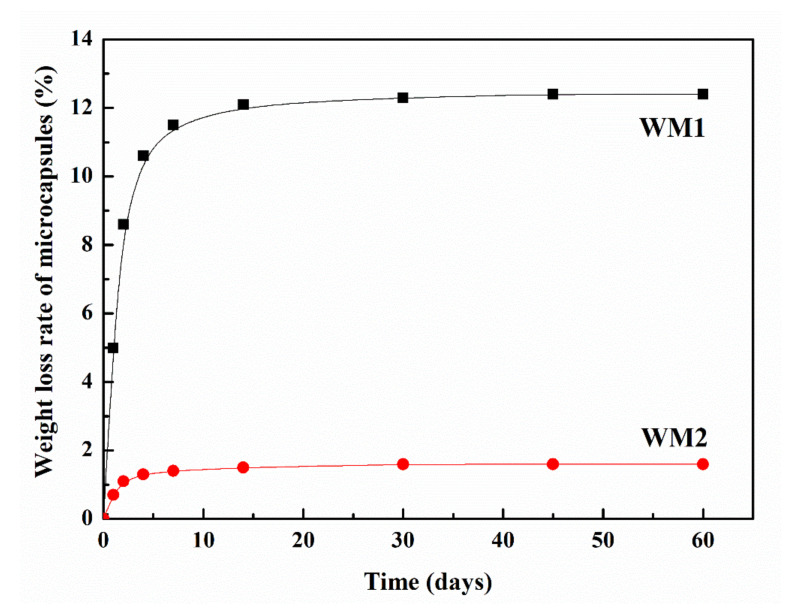
Weight loss rate of microcapsules.

Figure 3 describes the weight loss rate of microcapsules. The weight loss rates of WM1 at 1, 2, 4, 7, 14, 30, 45 and 60 days were 5%, 8.6%, 10.6%, 11.5%, 12.1%, 12.3%, 12.4% and 12.4%, respectively. As seen in Figure 3, the weight loss rate of WM1 rose rapidly from 1 to 14 days and then leveled off. However, the weight loss rates for WM2 were 0.7%, 1.1%, 1.3%, 1.4%, 1.5%, 1.6%, 1.6% and 1.6% at 1, 2, 4, 7, 14, 30, 45 and 60 days, respectively. For WM2, there was a small increase in the weight loss rate over a period of 4 days. After 60 days, the weight loss rate of WM2 was only 1.6%, which was much less than that of WM1 (12.4%), and the results indicated that WM2 was better compacted than WM1. This is due to the fact that after adding nano-CaCO_3_ to ceresine wax, the shell of microcapsules (nano-CaCO_3_/ceresine wax) is denser, and the microdefects on the surface are filled, which improves the encapsulation ability of E-44 epoxy resin, thus its weight loss rate decreases.

### 3.3. Particle Size Distributions

**Figure 4:**
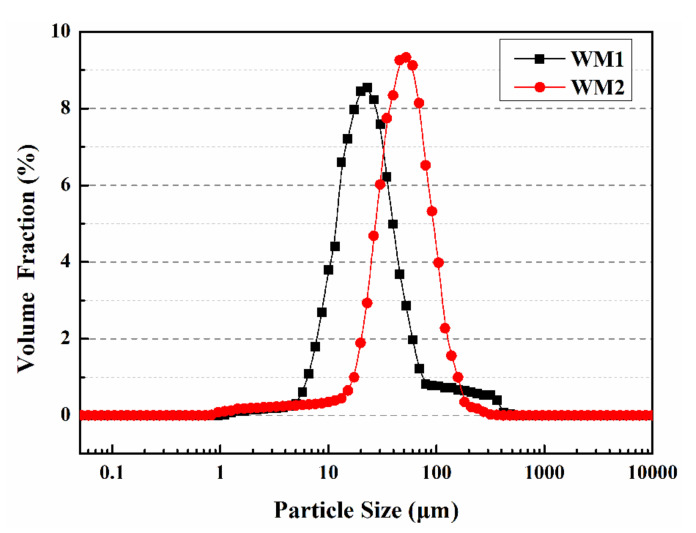
Particle size distribution of microcapsules.

The particle size distribution of microcapsules is shown in Figure 4. The particle size distribution of WM1 ranges from 1 µm to 80 µm. The particle size distribution of WM2 ranges from 20 µm to 120 µm, mainly from 40 µm to 60 µm. Table 6 shows the particle size values of D10, D50 and D90 of microcapsules, where the average particle sizes of WM1 and WM2 are 23 µm and 52 µm, respectively. This is due to the fact that the ceresine wax has a lower viscosity, which makes it easier to disperse during the mixing process. As a result, the particle size of WM1 became smaller under the action of shear force after the addition of coolant. The addition of nano-CaCO_3_ increases the viscosity of the shell mixture and reduces its ability to deform under shear force, thus the mixture is not easily dispersed during the mixing process for the preparation of microcapsules, and the particle size of the microcapsules increases at a constant stirring speed [25].

### 3.4. Morphologies of Microcapsules

**Figure 5:**
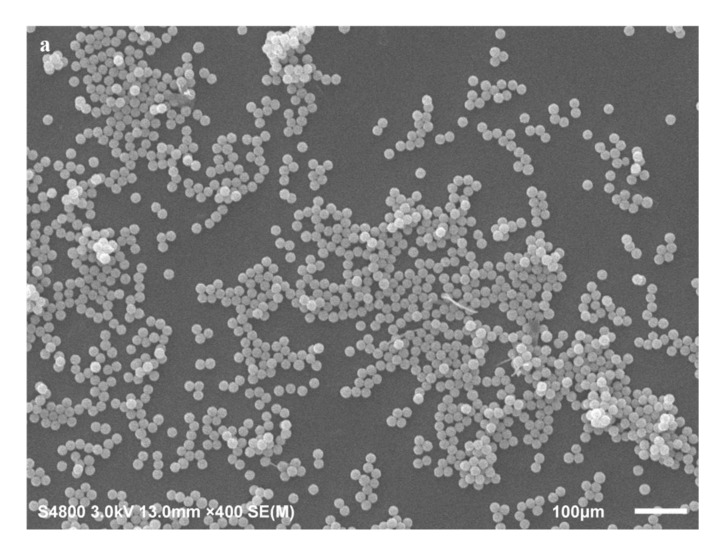

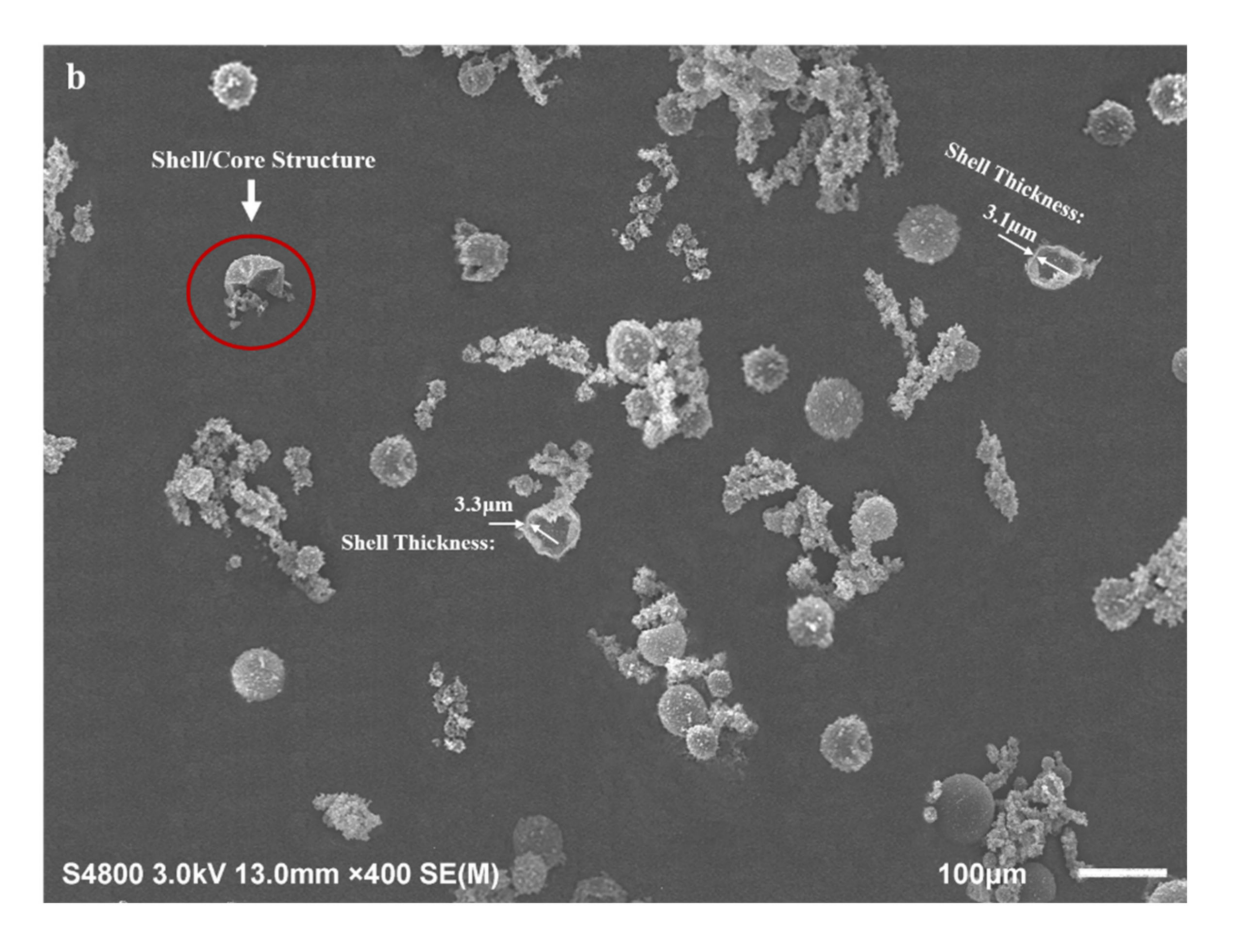
SEM images of microcapsules: (**a**) WM1, (**b**) WM2.

The surface morphology and internal structure of the microcapsules can be seen in Figure 5. As shown in Figure 5a, WM1 has a relatively smooth surface with a regular spherical shape and a particle size distribution of about 20 µm. Compared with WM1, it can be seen in Figure 5b that the surface of WM2 is rougher and the particle size is larger, mainly distributed around 50 µm. The reason is that the viscosity of ceresine wax is lower, the dispersion is better after heating and melting, and the microcapsules with smaller particle size and smoother surface are easily formed under the action of stirring force (WM1). When the nano-CaCO_3_ was added to the ceresine wax, the viscosity of the mixture increased, the dispersibility became poor, and it was not easily deformed under the action of the stirring bar shear force, resulting in a rough surface and larger particle size of the microcapsules. In addition, the observation of the ruptured WM2 in Figure 5b revealed that WM2 has a very obvious shell/core structure, in which the shell thickness of microcapsules with a diameter of 50 µm is 3.1 µm and that of microcapsules with a diameter of 52 µm is 3.3 µm, and the diameter/shell thickness ratio of microcapsules is about 17/1, which means that the shell of WM2 is very thin and can be used to encapsulate E-44 epoxy resin to provide an ideal storage space.

### 3.5. FTIR Analysis

**Figure 6:**
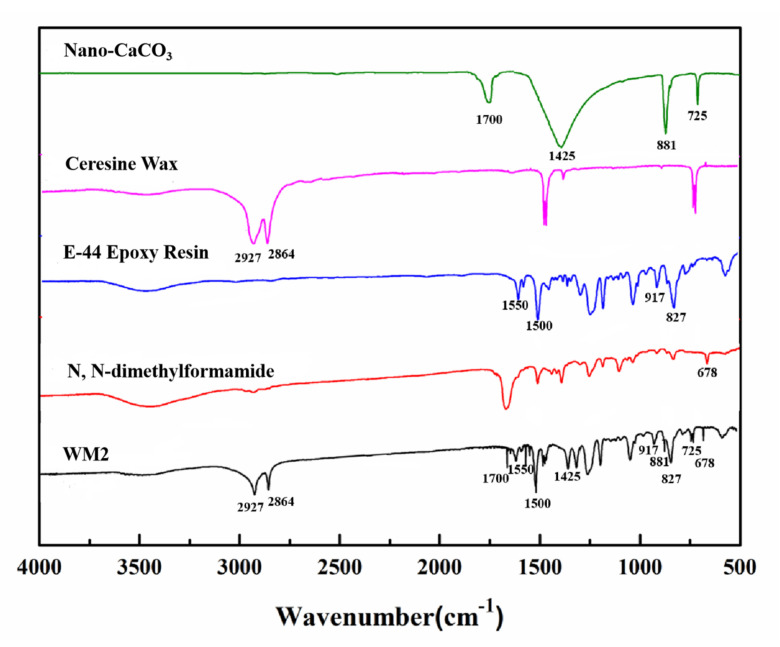
FTIR spectra of nano-CaCO3, ceresine wax, E-44 epoxy resin, N, N-dimethylformamide and WM2.

Figure 6 depicts the FTIR spectra of the nano-CaCO_3_, ceresine wax, E-44 epoxy resin, *N*,*N*-dimethylformamide and WM2. The characteristic peak located at 1700 cm^−1^ is the stretching vibration peak of C=O in nano-CaCO_3_, the characteristic peak at 1425 cm^−1^ is the antisymmetric stretching vibration peak of C–O in CO_3_^2−^, the peak at 881 cm^−1^ is the out-of-plane deformation vibration peak of CO_3_^2−^, and the in-plane deformation vibration peak of O–C–O is at 725 cm^−1^. The 2927 cm^−1^ and 2864 cm^−1^ characteristic peaks are from symmetric stretching vibrations and asymmetric stretching vibrations of –CH_2_- and –CH_3_ groups in ceresine wax, respectively. The peaks at 1550 cm^−1^ and 1500 cm^−1^ are characteristic peaks of stretching vibration of aromatic C–C and symmetric bending absorption peak of dimethyl in epoxy resin. The peaks at 917 cm^−1^ and 827 cm^−1^ are absorption peaks of C–O and C–O–C in ethylene epoxide group. The characteristic peak at 678 cm^−1^ is the absorption peak of amide I band, which implies that a small amount of *N*,*N*-dimethylformamide is encapsulated in WM2. The presence of characteristic peaks corresponding to nano-CaCO_3_, ceresine wax, E-44 epoxy and N, N-dimethylformamide can be found in the FTIR spectra of WM2. Combining the SEM and FTIR results, it can be concluded that E-44 epoxy was successfully encapsulated in the nano-CaCO_3_/ceresine wax composite shell.

### 3.6. Micromechanical Properties

Table 7 shows the elastic modulus and hardness of the microcapsules characterized by nanoindentation tests. The elastic modulus of WM1 and WM2 are 0.55 GPa and 2.02 GPa, respectively. The hardness of WM1 and WM2 are 4.89 MPa and 72.54 MPa, respectively. The results show that the elastic modulus and hardness of WM2 are higher than those of WM1. The mechanical properties of microcapsules are mainly determined by their shell materials. After the nano-CaCO_3_ is blended with ceresine wax, the nano-CaCO_3_ acts as a physical cross-linking point, and the molecular chains of ceresine wax will be wrapped around the nano-CaCO_3_ particles. When one of the ceresine wax molecular chains is under stress, the nano-CaCO_3_ can transfer the stress to the other molecular chains, so that the stress is dispersed. Even if the network chain is broken at one place, due to the “cross-linking-like” effect of nano-CaCO_3_, the other molecular chains can still withstand the stress and do not rapidly endanger the whole, reducing the possibility of fracture and playing a strengthening role [27].

**Figure 7:**
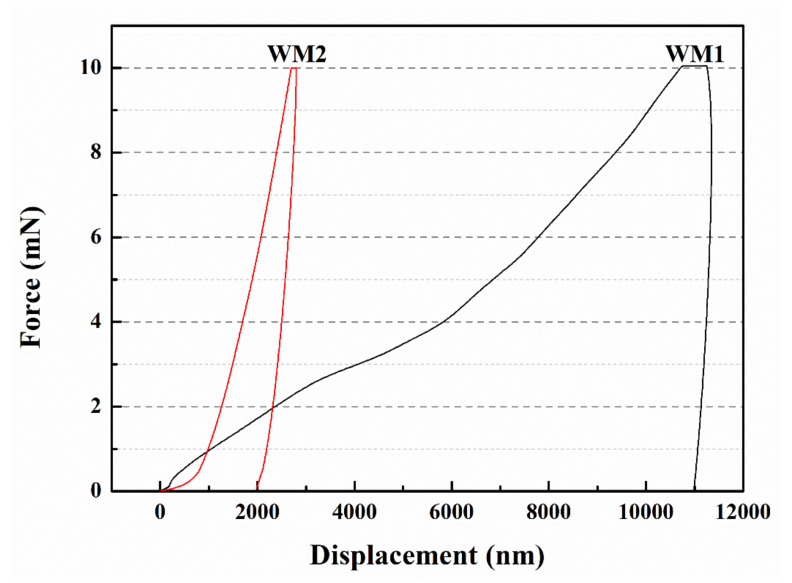
Force-displacement curves of microcapsules.

Figure 7 demonstrates the force-displacement curves of the microcapsules. A 1980 nm displacement for WM2 and 10,998 nm displacement for WM1 indicates that the displacement of WM2 is much smaller than that of WM1. The ability of microcapsules to resist plastic deformation induced by pressure is determined by the elastic modulus and hardness. In general, materials with high elastic modulus and hardness are able to produce smaller elastic deformation when subjected to external forces [28]. The addition of nano-CaCO_3_ to the microcapsule shells significantly increased their elastic modulus and hardness, thus reducing the displacement of the microcapsules.

### 3.7. Compressive Strength Recovery Rate

Table 8 shows the compressive strength of the mortar after 28 days of standard curing. The compressive strengths of CCM0, CCM1 and CCM2 were 31.2 MPa, 40.6 MPa and 35.5 MPa, respectively. Compared with CCM0, the compressive strengths of CCM1 and CCM2 increased by 30.1% and 13.8%, respectively, indicating that an appropriate dosage of microcapsules can significantly improve the compressive strength of the mortar. This is due to the fact that the microcapsules can fill internal pores of the mortar and thus improve the compressive strength of the mortar [29]. The compressive strength of CCM1 was higher than that of CCM2 because the particle size of WM1 was smaller than that of WM2, which can better fill the internal pores of the mortar and reduce the microdefects inside the mortar [30].

**Figure 8:**
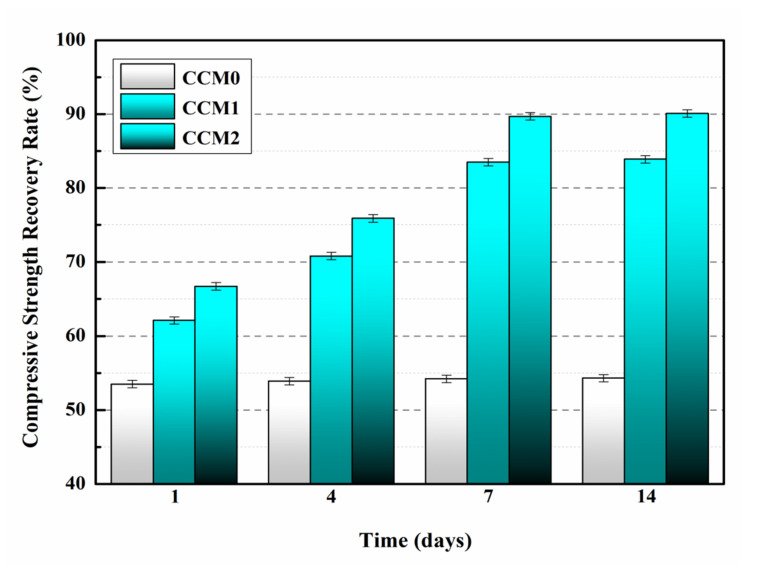
Compressive strength recovery rates of mortars.

Figure 8 depicts the compressive strength recovery rate of the mortar as the self-healing time increases. It can be found that the compressive strength recovery rate of CCM0 did not change with increasing self-healing time, a phenomenon that indicates that the intrinsic self-healing ability of cement-based materials is unable to self-heal the pre-damaged CCM0 after 14 days.

**Figure 9:**
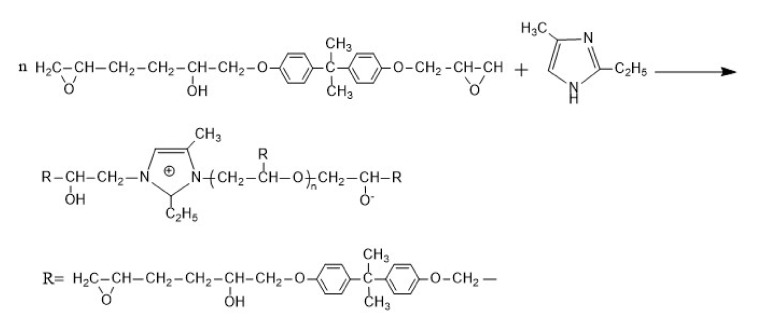
Curing process of E-44 epoxy resin and 2-ethyl-4-methylimidazole.

It can be seen from Figure 8 that the compressive strength recovery rates of CCM1 and CCM2 were significantly higher compared with CCM0. The compressive strength recovery rates of CCM1 and CCM2 rose gently during the self-healing time of 1–4 days, while they increased rapidly during the self-healing time of 4–7 days and remained basically constant for 7–14 days, which indicates that the self-healing process of microcapsules on pre-damaged mortar was completed after 14 days. This is because microcracks appear in the mortar after being pre-loaded, and the stresses at the tips of these cracks can rupture the shell of the microcapsules. The E-44 epoxy resin is released from the microcapsules and flows into the microcracks, where it comes into contact with the 2-ethyl-4-methylimidazole pre-mixed in the mortar to produce curing products that self-heal the microcracks. Figure 9 shows the chemical reaction process of E-44 epoxy resin and 2-ethyl-4-methylimidazole.

After 14 days of self-healing, the compressive strength recovery rates of CCM0, CCM1 and CCM2 were 54.3%, 83.9% and 90.1%, respectively. Compared with CCM0, the compressive strength recovery rates of CCM1 and CCM2 increased by 54.5% and 65.9%, respectively. The results indicate that WM2 has better self-healing ability for pre-damaged mortar. This is mainly due to the higher initial core content and better compactness of WM2 than WM1 (the microcapsules used in the self-healing experiments were prepared 60 days in advance), which ensured that the self-healing mortar had more healing agent, resulting in a higher compressive strength recovery rate for CCM2 than CCM1. In addition, the micromechanical properties of WM2 were better than WM1, which reduced the risk of rupture during mortar mixing, and the E-44 epoxy resin was not lost due to microcapsule damage during mortar preparation, thus improving the self-healing ability of the pre-damaged mortar.

### 3.8. Pore Size Distribution of Mortars

It has been shown that pores larger than 0.1 µm in diameter in cement-based materials will adversely affect mechanical properties and impermeability, and they are considered harmful pores [31]. Therefore, this section will focus on the proportion of pores larger than 0.1 µm in diameter in mortars.

**Figure 10:**
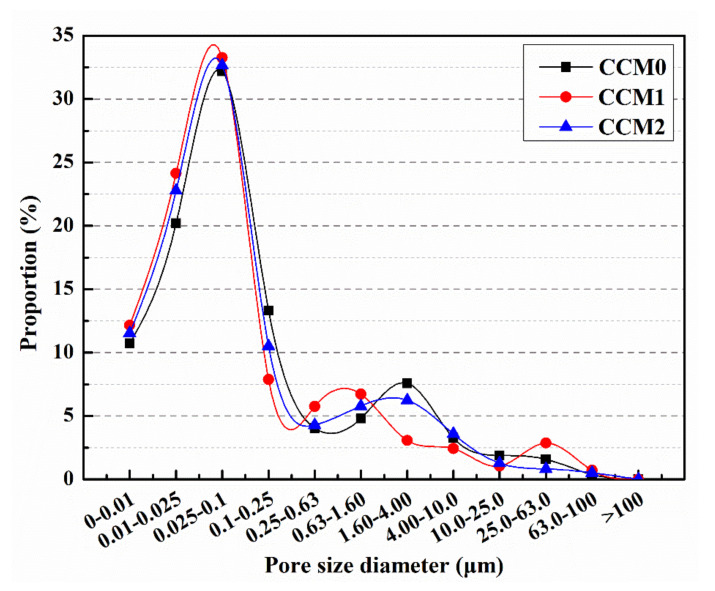
Pore size distribution of mortars.

Figure 10 illustrates the pore size distribution of the mortar after 28 days of standard curing. The proportion of harmful pores for CCM0, CCM1 and CCM2 was 36.89%, 30.44% and 33.04%, respectively. Compared to CCM0, the proportion of harmful pores of CCM1 and CCM2 declined by 17.5% and 10.4%, respectively. This indicates that the proportion of harmful pores was reduced, and the compactness was improved by adding microcapsules to the mortar. This is due to the fact that mortar is formed by mixing two materials of different particle sizes, cement and sand, with water. After formation, a certain number of pores exist in the mortar. By adding microcapsules to the mortar during the preparation process, some pores and microdefects in the mortar can be filled, at which point the internal structure of the mortar becomes more compact and the proportion of its harmful pores is reduced [32].

**Figure 11:**
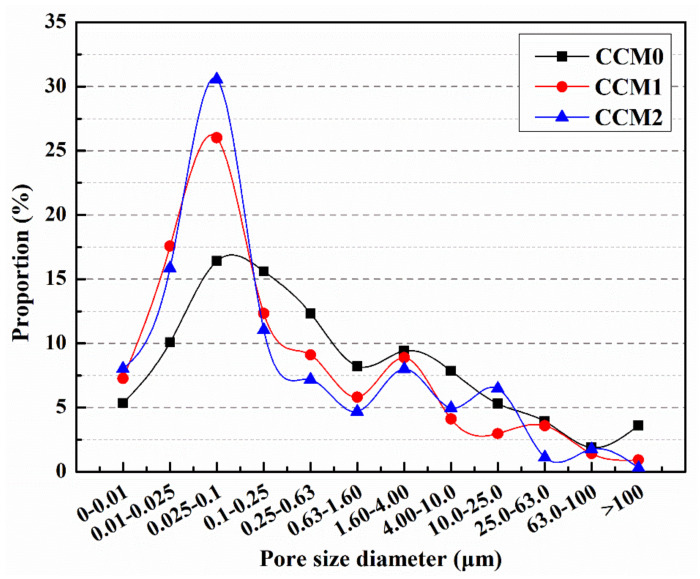
Pore size distribution of mortars after 14 days of self-healing.

Figure 11 shows the pore size distribution of the pre-damaged mortar after 14 days of self-healing. The proportion of harmful pores was 68.17%, 49.15% and 45.54% for CCM0, CCM1 and CCM2, respectively. It was found that the mortar with microcapsules had significantly lower proportion of harmful pores after self-healing. This is because the pre-load causes internal cracking of the mortar, while the microcracks encounter the microcapsules during the extension process. The shell materials of microcapsules are mainly thermoplastic materials (ceresine wax, nano-CaCO_3_/ceresine wax), which are easy to rupture under the stress, and the released E-44 epoxy resin cures in contact with 2-ethyl-4-methylimidazole to generate healing products that fill the microcracks and reduce the proportion of harmful pores in the mortar.

### 3.9. Chloride Ion Diffusion Coefficient Recovery Rates of Mortars

The chloride ion diffusion coefficients of the mortar after standard curing for 28 days are shown in Table 9. The chloride ion diffusion coefficient of CCM0 was 16.99 × 10^−12^ m^2^/s. After adding 4% cement weight of WM1 or WM2 to the mortar, the chloride diffusion coefficients of CCM1 and CCM2 were 14.68 × 10^−12^ m^2^/s and 15.77 × 10^−12^ m^2^/s, respectively. Compared with CCM0, the chloride ion diffusion coefficients of CCM1 and CCM2 decreased by 13.6% and 7.2%, respectively. This is because some small pores appear inside the mortar after molding, and adding the appropriate dosage of microcapsules can fill some pores, increase the compactness of the mortar, reduce its chloride ion diffusion coefficient and thus improve the impermeability of the mortar [33]. Since the particle size of WM1 is smaller than that of WM2, it can better fill the pores and microdefects inside the mortar, hinder the flow transmission of chloride ions and increase the compactness of mortars, which leads to a more obvious improvement in the impermeability of the mortar [34].

**Figure 12:**
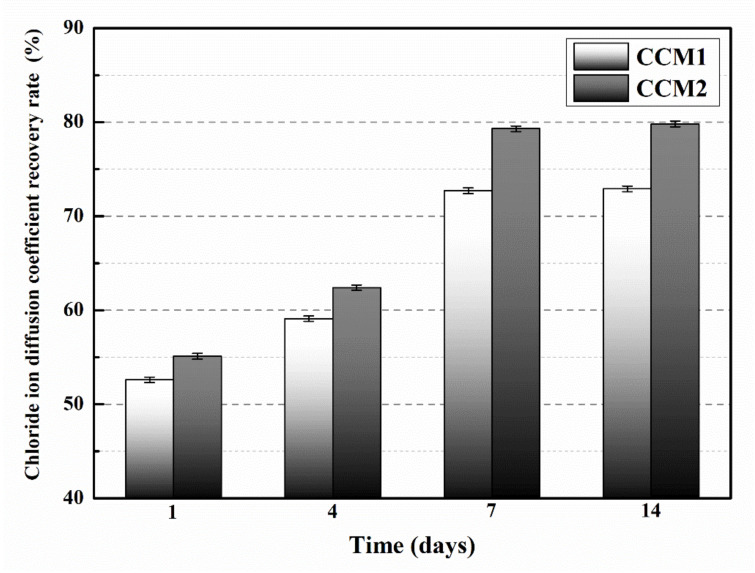
Chloride ion diffusion coefficient recovery rates of mortars.

Figure 12 shows the chloride ion diffusion coefficient recovery rates of mortars after different self-healing periods. After 1, 4, 7 and 14 days of self-healing, the chloride ion diffusion coefficient recovery rates were 52.6%, 59.1%, 72.7% and 72.9% for CCM1, while the chloride ion diffusion coefficient recovery rates for CCM2 were 55.1%, 62.4%, 79.3% and 79.8%, respectively. This result is due to the different microcapsules incorporated in the mortar. From the experimental results in Section 3.1 and Section 3.2, it can be seen that WM2 had a higher core content and less weight loss after 60 days of placement, which means that more E-44 epoxy resin is involved in the self-healing reaction. Therefore, more healing products can be produced, reducing the channels for chloride ion transport in the mortar, resulting in a higher chloride ion diffusion coefficient recovery rate for CCM2 than CCM1.

### 3.10. Self-Healing of Surface Cracks

**Figure 13:**
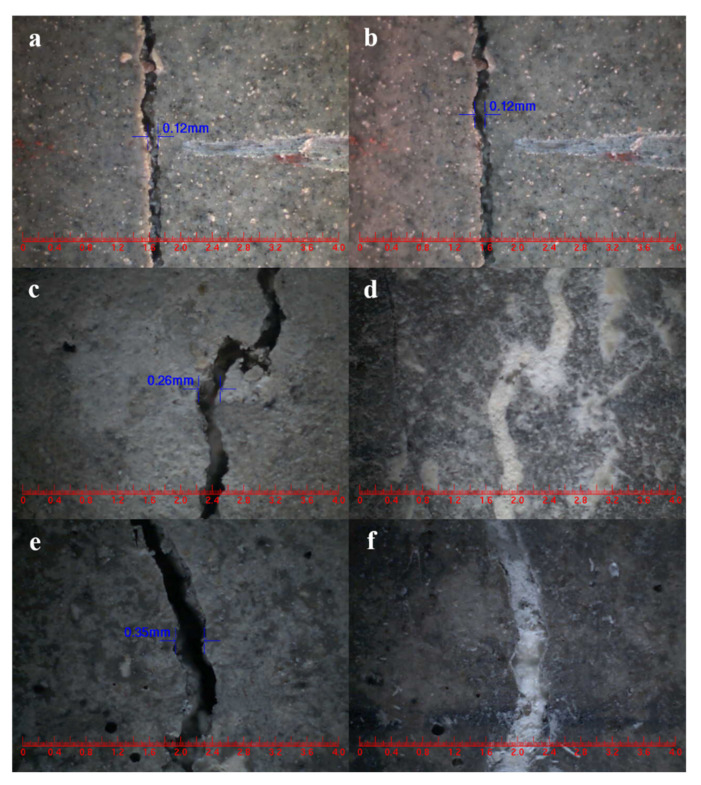
Surface crack self-healing of mortars. (**a**) CCM0 before self-healing, (**b**) CCM0 after 3 days of self-healing, (**c**) CCM1 before self-healing, (**d**) CCM1 after 3 days of self-healing, (**e**) CCM2 before self-healing, (**f**) CCM2 after 3 days of self-healing.

Self-healing cement-based materials have been developed to automatically heal cracks as they occur. The purpose of this section aims to provide convincing evidence for the self-healing ability of cement-based materials containing microcapsules. The crack widths of the mortar before and after self-healing were measured. From Figure 13a,b, the surface crack of CCM0 with an initial width of 0.12 mm did not change its width after 3 days, indicating that the intrinsic self-healing ability of the cement-based material was unable to heal its surface crack. On the contrary, surface cracks of CCM1 and CCM2 with initial widths of 0.26 mm and 0.35 mm could completely self-heal after 3 days, as obtained from Figure 13d,f. This indicates that microcapsules can effectively self-heal the cracks in the mortar. After 3 days, the self-healing width of CCM2 is wider than that of CCM1 after 3 days. This is due to the higher compactness and initial core content of WM2 than WM1, which leads to a wider crack self-healing width of CCM2. Meanwhile, WM2 has good micromechanical properties, which avoids the risk of fracture during the mortar mixing and prevents the loss of E-44 epoxy resin, which also facilitates the healing of wider cracks.

## 4. Conclusions

In this paper, microcapsules with nano-CaCO_3_/ceresine wax composite shell and E-44 epoxy resin healing agent were prepared via the melt condensation method. We measured the core content, compactness, particle size distribution, morphology, chemical structure and micromechanical properties of microcapsules. The microcapsules were then mixed into the mortar, and the self-healing ability of the mortar containing microcapsules was investigated. The following conclusions can be drawn from this study:

(1) The results indicated that the addition of nano-CaCO_3_ to ceresine wax could further improve the encapsulation ability, mechanical properties and compactness of microcapsules. The core content, elastic modulus, hardness and weight loss rate (60 days) of WM2 were 80.6%, 2.02 GPA, 72.54 MPa and 1.6%, respectively.
(2) The particle size distribution of WM2 mainly ranged from 40 µm to 60 µm. It could be seen from the SEM images that the surface of WM2 is rough and can form a good bond with the cement matrix. FTIR spectra shows that TDI has been successfully encapsulated in the nano-CaCO_3_/ceresine wax composite shell.
(3) After 14 days of self-healing, the compressive strength recovery rate, proportion of harmful pores and chloride ion diffusion coefficient recovery rate of CCM2 were 90.1%, 45.54% and 79.8%, respectively. The surface crack with an initial width of 0.35 mm in CCM2 self-healed after 3 days. The results indicate that the microcapsules can rapidly self-heal the surface cracks of the mortar and fill its internal harmful pores, thus recovering the mechanical properties and impermeability of the pre-damaged mortar.

## Figures and Tables

**Table 1:** Chemical composition of cement (%).

CaO	SiO_2_	Al_2_O_3_	MgO	Fe_2_O_3_	SO_3_	Other
58.44	21.82	6.58	2.34	4.02	2.35	3.45

**Table 2:** Particle size distribution of the standard sand *.

Square Mesh Size (mm)	Cumulative (%) Retained
0.08	99 ± 1
0.16	87 ± 5
0.50	68 ± 5
1.00	33 ± 5
1.60	7 ± 5
2.00	0

* More information about standard sand is on the website. (https://www.standard-sand.com/en/standard-sand-cen-en-196-1-2/ (accessed on 13 May 2020).

**Table 3:** Preparation parameters and shell/core weight ratios of microcapsules.

Microcapsule	Shell Material Used by Weight Ratio	Core Material Used by Weight Ratio	Heating Temperature	Stirring Speed	Viscosity of Shell at Heating Temperature
WM1 *	ceresine wax, 45	E-44 epoxy resin diluted with DMF, 55	130 °C	1200 rpm	5.5 mPa·s
WM2 *	ceresine wax, 40; nano-CaCO_3_, 5	E-44 epoxy resin diluted with DMF, 55	130 °C	1200 rpm	35 mPa·s

WM1 *: ceresine wax containing E-44 epoxy resin microcapsule; WM2 *: nano-CaCO_3_/ceresine wax containing E-44 epoxy resin microcapsule.

**Table 4:** Formulations of mortars according to the weight ratio.

Mortar	Cement	Water	Sand	Microcapsules
CCM0	100	50	300	0
CCM1	100	50	300	4 (WM1)
CCM2	100	50	300	4 (WM2)

**Table 5:** Core content of microcapsules.

Microcapsule	Core Content
WM1	75.4%
WM2	80.6%

**Table 6:** Particle sizes of microcapsules.

Microcapsule	D10 Values/µm	D50 Values/µm	D90 Values/µm
WM1	10	23	50
WM2	25	52	115

The D10 values, D50 values and D90 values indicate that the volume diameter of the microcapsules is less than 10%, 50% and 90% of these values, respectively.

**Table 7:** Elastic modulus and hardness of microcapsules.

Microcapsules	Elastic Modulus (GPa)	Hardness (MPa)
WM1	0.55	4.89
WM2	2.02	72.54

**Table 8:** Compressive strength of mortars.

Mortars *	Compressive Strength (after 28 Days of Standard Curing)
CCM0 *	31.2 MPa
CCM1 *	40.6 MPa
CCM2 *	35.5 MPa

* CCM0: control mortar; * CCM1: the mortar containing WM1; * CCM2: the mortar containing WM2.

**Table 9:** Chloride ion diffusion coefficients of mortars.

Mortars	Chloride Ion Diffusion Coefficient (after 28 Days of Standard Curing)
CCM0	16.99 × 10^−12^ m^2^/s
CCM1	14.68 × 10^−12^ m^2^/s
CCM2	15.77 × 10^−12^ m^2^/s
CCM60 *	44.02 × 10^−12^ m^2^/s

* CCM60: the control mortar under 60%f_a_ pre-load.

## Data Availability

Data is contained within the article and Supplementary Materials.

## Supplementary Materials

The following are available online at https://www.mdpi.com/article/10.3390/nano12020197/s1. Nanoindentation test and Pore size Distribution.
