# Supplementary material for: Preparation and Characterization of Nano-CaCO3/Ceresine Wax Composite Shell Microcapsules Containing E-44 Epoxy Resin for Self-Healing of Cement-Based Materials
Source: Nanomaterials (Basel). 2022 Jan 7;12(2):197. doi: 10.3390/nano12020197 (PMC8778703; doi:10.3390/nano12020197)
Supplement: Supplementary file 1 [file nanomaterials-12-00197-s001.zip › nanomaterials-1478299-supplementary.pdf]

nanomaterials

Supplementary Materials: Preparation and Characterization of Nano-$CaCO_3$/Ceresine Wax Composite Shell Microcapsules Containing E-44 Epoxy Resin for Self-Healing of Cement-Based Materials

Wei Du [1,2,*], Erwang Li [1] and Runsheng Lin [3]

Nanoindentation test

The microcapsules used for nanoindentation testing were prepared as follows: First, the embedding agent (cold mount resin) was poured into a mold of Φ2.5 cm, and then microcapsules were added to it. After the embedding agent hardened, the surface of the microcapsules embedded in the cold mount resin was polished into a smooth sphere. Nanoindentation tests were performed by a Triboindenter (TI-900, Hysitron, Hysitron, MN, USA) and fitted with a Berkovich tip. The indenter was in contact with the sample surface and had a calibrated trapezoidal load function defined as a loading rate of 20.00 mN/min, a holding time of 5 s at a maximum load of 10 mN, and an unloading rate of 20.00 mN/min. These mechanical values were recorded and plotted on a graph to create a load-displacement curve. The contact stiffness (S) was defined as the slope of the unloading curve and was calculated by Equation (1).

$$S=\frac{dp}{dh}=\frac{2}{\pi}E_r\sqrt{A} \quad (1)$$

Hardness (H) was calculated by Equation (2):

$$H=\frac{P_{max}}{A} \quad (2)$$

where A is the projected contact area at peak load P_{max} and E_r is the reduced elastic modulus.

Pore size distribution

The pore size distribution of the mortar was investigated using a nuclear magnetic resonance spectrometer (MesoMR23, Suzhou Newman Analytical Instrument Co., Ltd, Suzhou, China) based on the relationship between the relaxation time of the hydrogen spectrum and the pore size of water molecules in the specimen. In this experiment, the pore size distribution of mortars subjected to pre-damage (60%f_a) and self-healing (25°C, 50% RH) after 14 days was measured. Before testing, the specimens were wiped clean and saturated in a water vacuum for 24 h. The resonant frequency was 23.04 MHz, the magnet temperature was 32.00 ± 0.02°C, and the probe diameter was 25 mm. The pore diameter was computed by Equation (3).

$$\frac{1}{T_2}=\rho\left(\frac{S}{V}\right)_{pore} \quad (3)$$

where T_2 is the relaxation time of water in the pore (ms), ϱ is the surface relaxation rate (70 μm/ms), and $(S/V)_{pore}$ is the pore surface area to volume ratio.
